# Impact of Medically Tailored Meals on Clinical Outcomes Among Low-Income Adults with Type 2 Diabetes: A Pilot Randomized Trial

**DOI:** 10.1007/s11606-024-09248-x

**Published:** 2024-12-13

**Authors:** Jeanne M. Clark, May Thu Thu Maw, Kathy Pettway, Geetanjali Chander, Susan Elias, Sam Zisow-McClean, Nisa M. Maruthur, Raquel C. Greer

**Affiliations:** 1 Division of General Internal Medicine, Department of Medicine, Johns Hopkins School of Medicine, Baltimore, MD USA; 2 Johns Hopkins Brancati Center for the Advancement of Community Care, Baltimore, MD USA; 3 Department of Medicine, Rutgers Robert Wood Johnson Medical School, New Brunswick, NJ USA; 4 Department of General Internal Medicine, University of Maryland - Capital Region Medical Center, Largo, MD USA; 5 Johns Hopkins Health Plans, Hanover, MD USA; 6 Division of General Internal Medicine, Department of Medicine, University of Washington School of Medicine, Seattle, WA USA; 7 Moveable Feast, Baltimore, MD USA

**Keywords:** Type 2 diabetes mellitus (T2DM), Health disparities, Medically tailored meals (MTM), Medical nutrition therapy (MNT), Pilot randomized trial

## Abstract

**Background:**

Adults with type 2 diabetes (T2DM) and adverse social determinants of health experience barriers to healthful eating, and achieve poorer glycemic control and clinical outcomes.

**Objective:**

To examine the impact of medically tailored meals (MTM) with medical nutrition therapy (MNT) on clinical outcomes among adults with DM.

**Design:**

Pilot randomized controlled trial.

**Participants:**

English-speaking adults with DM and hemoglobin A1c (A1c) levels > 8% insured by Maryland Medicaid plans.

**Intervention:**

The treatment group received home delivery of 12 medically tailored, frozen meals and a fresh produce bag weekly for 3 months, and individual calls with a registered dietitian monthly for 6 months in addition to usual care. The control group received usual care. Outcomes were change from baseline to 6 months in A1c (primary), body mass index (BMI), blood pressure, food insecurity, and diabetes-related quality of life, knowledge, and self-efficacy (secondary).

**Key Results:**

We randomized 74 adults; 77% completed data collection. The mean age was 48 years, 40% were male, 77% were Black, and the mean A1c was 10.3%. Eighty-six percent of meals were delivered, and on average 4.8 nutrition visits were completed. At 6 months, both groups had similar improvements in A1c (− 0.7 vs. − 0.6%); the control group reported more favorable changes in diabetes medications. Changes in systolic blood pressure and BMI at 6 months did not differ between groups. Diabetes-related quality of life, knowledge, and self-efficacy improved modestly, but not differently by group. Food insecurity decreased significantly from baseline to 3 months in the intervention (53 to 17%) compared to control (48 to 44%; *p* < 0.05), which lessened but remained significant at 6 months.

**Conclusions:**

Recruitment and retention of an at-risk group of adults with DM was feasible. Intervention uptake was good but did not improve clinical outcomes. More comprehensive and clinically integrated interventions are likely needed to achieve significant clinical benefits.

**ClinicalTrials.gov Registration:**

NCT04034511.

**Supplementary Information:**

The online version contains supplementary material available at 10.1007/s11606-024-09248-x.

## INTRODUCTION

Type 2 diabetes mellitus (T2DM) affects more than 38 million adults in the USA and is the leading cause of blindness, kidney failure, and non-traumatic amputation.^1^ The total estimated cost of DM in the USA in 2017 was $327 billion, accounting for 1 in 4 US health care dollars.^2^ DM disproportionately affects those from racial and ethnic minority groups; the prevalence is 81% and 70% higher among Black and Hispanic individuals compared to non-Hispanic White individuals, respectively.^3^ It is also nearly 200% higher in those with a high school education or less, and with lower socioeconomic status.^4,5^

Tracking by the US Diabetes Surveillance System demonstrates clear disparities in DM complications for Black compared to White persons, with hospitalizations for hyper- and hypoglycemia being 33 to 204% higher, end-stage kidney disease being 219% higher, and visual impairment being nearly 50% higher.^1^ Overall, Black persons are more than twice as likely to experience premature mortality related to DM than White persons. While fewer data are available on complication rates by socioeconomic status, persons with less than high school education are nearly 60% more likely to have DM-related visual impairment than those with more than high school education.^1^ Adults with low income also experience greater rates of diabetes-related complications and mortality.^6,7^ Limited published data also demonstrate disparities in diabetes care and complications among those insured with Medicaid.^8–10^

Dietary habits play an important role in achieving and maintaining glycemic control.^11^ However, adults with low income are often less able to adopt the necessary dietary changes to improve their glycemic control due to poor access to diabetes education as well as financial and environmental constraints that limit access to healthy foods.^12^ Persons with DM and food insecurity are more likely to skip medications doses, have higher hemoglobin A1c (A1c) levels, and are less likely to receive guideline-concordant care.^13^ Nutrition interventions that target barriers to healthy dietary habits among adults with low income and DM may have a profound impact on improving glycemic control. Evidence suggests the provision of medically tailored meals (MTM) may be beneficial in improving health outcomes and health care costs among socially disadvantaged adults with chronic illnesses.^14–17^ The provision of home-delivered MTM in addition to individualized medical nutrition therapy (MNT) is a promising approach to improve dietary habits in under-resourced populations with T2DM; however, randomized controlled studies specifically exploring the benefits of MTM and MNT among adults with uncontrolled T2DM on clinical outcomes such as HbA1c are lacking.

We conducted a pilot randomized controlled clinical trial to determine the feasibility and impact of MTM for 3 months and MNT for 6 months on health-related outcomes and health care costs among adults with low income and T2DM. We hypothesized that the intervention would improve levels of A1c (primary outcome), as well as changes in quality of life, self-efficacy with diabetes self-management, blood pressure, body weight, food insecurity, diet quality, and total medical costs and utilization.

## METHODS

### Study Design

We conducted a single-site, randomized controlled pilot trial to determine the feasibility and estimate the effect size of an intervention which included 3 months of MTM and 6 months of MNT. This pilot study is reported according to CONSORT guidelines—pilot trial extension and is registered on ClinicalTrials.gov (NCT-NCT04034511).

### Study Population

We recruited English-speaking adults aged 18 and over who were enrolled in and identified by a Maryland Medicaid Insurance plan (91%) or from a clinical database (8.5%) or by direct referral from a provider (< 1%) as having T2DM with the most recent A1c level greater than 8.0%. Participants were required to have a refrigerator/freezer in the home to store meals and be staying in the area for the next 12 months. Individuals with medical conditions requiring special or additional dietary requirements (e.g., advanced chronic kidney disease with eGFR < 30 or current pregnancy), swallowing difficulties, significant food allergies and active substance use disorder were excluded.

Participants were recruited via mailed study flyer with opt-in and opt-out options. Ten days after the mailing, participants were called by Medicaid plan staff to assess interest and confirm basic eligibility. Participants were then connected directly to the study team who conducted full assessment of eligibility over the phone, followed by in-person visit. Partway through the study, we additionally took direct referrals from primary care clinicians who could contact the study team for eligibility assessment.

The study began in January 2020, with data collection completed in April 2023. Ten participants were enrolled and randomized before the onset of the COVID-19 pandemic and had both their intervention and data collection affected by temporary cessation of all study activities imposed by the IRB. Before resuming in September 2020, COVID safety mechanisms were put into place, and the MNT intervention was switched to phone call only (initially there was the potential for home visit assessments).

### Interventions

Participants in this parallel arm designed trial were randomly assigned 1:1 to the active intervention with usual care or usual care alone.

The active intervention was a combination of medically tailored meals (MTM), and medical nutrition therapy (MNT). MTM was managed by Moveable Feast, a Baltimore-headquartered community-based organization, and included 12 medically tailored frozen meals and a fresh produce bag (containing five additional servings) weekly for 3 months. These meals were tailored for a population with T2DM specifically based on the American Heart Association diet, providing 60 g of carbohydrates per meal. Participants were provided instructional materials on how to reheat the meals and how to add flavor to meals without added salt. The first delivery also included a bottle of house-made salt-free seasoning blend. MNT consisted of individual phone calls with a registered dietitian from Moveable Feast monthly for 6 months. These sessions were self-directed and included goal-setting and general education about nutrition, carbohydrates, physical activity, and diabetes-friendly dietary approaches. Usual care was not augmented by the study team in any way. All participants had Medicaid insurance and were encouraged to continue their routine visits with their primary care clinician and/or endocrinologist, who were free to make any changes to their patient’s medications or make other recommendations as they saw fit.

### Data Collection and Outcome Measures

Sociodemographic information was collected at baseline. Additionally, we collected comorbidities, dietary intake, food insecurity, quality of life, and self-efficacy using standardized questionnaires at baseline, 3 months, and 6 months. Weight, blood pressure, and A1c were also measured by the study team using standardized protocols at baseline, 3 months, and 6 months (Supplemental Table 3). Data collection was overseen by trained study team members and entered into Research Electronic Data Capture (REDCap)^18,19^ or the online ASA24 diet recall platform. In-person data was collected at the institution’s on-site clinical research unit. Health care utilization data, including costs and the standard of care A1c at 12 months, were collected by the Medicaid insurer and transferred to the study team at the end of the study.

The primary outcome was the between-group differences in the change in A1c from baseline to 6 months. A point-of-care A1c was measured during the study visit using the Abbott Afinion AS100 Analyzer machine. We chose 6 months to assess whether the effects of the 3-month MTM were sustained and to evaluate the cumulative impact of the MTM and MNT.

Secondary outcomes included (1) between-group differences in the change in A1c from baseline to 12 months, (2) diabetes-related health care utilization and health care costs for 6 and 11 months post enrollment compared to a similar period prior to enrollment; as well as between-group differences from baseline to 3 and/or 6 months in the change in: (3) the number, type, and dosages of diabetes-related medications, (4) quality of life from the Audit of Diabetes-Dependent Quality of Life (ADDQOL), (5) self-efficacy with diabetes self-management, using the Perceived Diabetes Self-Management Scale (PDSMS), (6) blood pressure, (7) body weight, (8) changes in food insecurity, as measured by USDA’s Household Food Security Questionnaire,^20^ from baseline to 3 months and to 6 months, and (9) change in diet quality at baseline from baseline to 3 months and to 6 months using the ASA24 diet recall and Healthy Eating Index 2015 Scores (HEI), both total and the 13 individual components.

### Statistical Analysis

We summarized baseline characteristics of participants using means for continuous variables and counts/percentages for categorical variables. Analyses of outcomes were limited to those with A1c data at baseline and 6 months; no imputation was used. We used paired *t*-tests to conduct difference-in-differences analyses of change in the primary and secondary outcomes from baseline to 3 or 6 months between the two groups. We examined within-group changes from baseline to 3, 6, or 12 months as applicable. We used a *p* value of 0.05 to indicate statistical significance for all analyses. We originally proposed a sample size of 100 participants (50 per arm), which would provide 80% power to detect a difference of 1.14% in A1c. However, being a pilot study, the main goal analytically was to get an estimate of the effect size of the intervention.

Cost data were categorized into total, inpatient, emergency room, pharmacy, and remaining costs (e.g., outpatient services). We constructed time windows of 6- and 11-months duration, with enrollment as time 0, and excluding the month of enrollment. Time windows thus included (1) − 6 months to time 0, (2) − 11 months to time 0, (3) + 1 month to + 7 months, and (4) + 1 month to + 12 months.

The study was reviewed and approved by the Johns Hopkins Institutional Review Board, as well as the Office of Internal Controls and Audit Compliance (IAC) of the Maryland Department of Health.

## RESULTS

### Study Population and Baseline Characteristics

**Figure 1:**
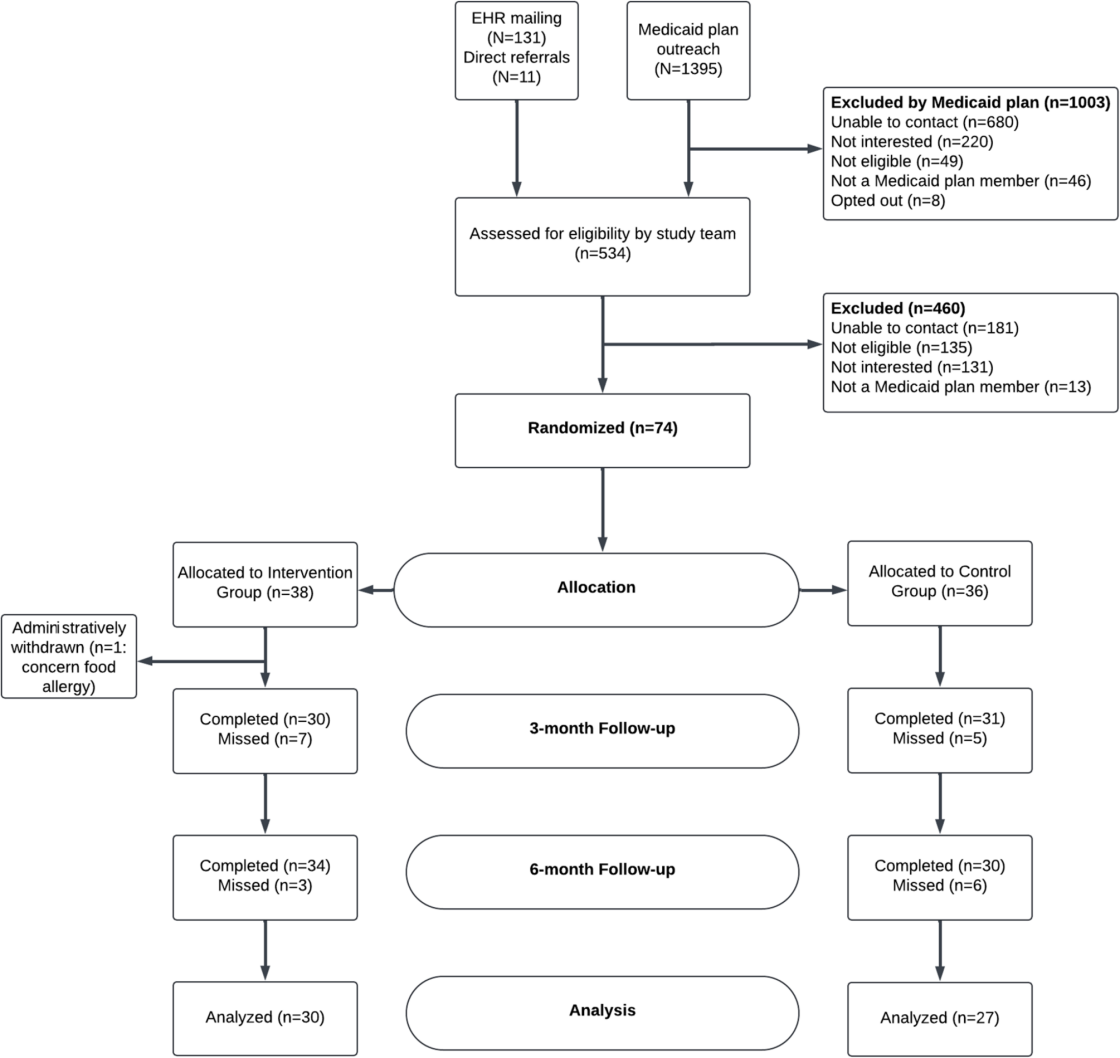
CONSORT diagram for the FEED study

Between January 2020 and March 2022, a total of 1537 adults were contacted about the study. The majority, 91% (1395 individuals), were enrollees in the partnering Medicaid plan with a diagnosis of T2DM to whom we distributed letters and brochures. We randomly allocated 74 individuals to participate in the study: 73 were from mailings and 1 came from a direct referral (Supplemental Table 1). During the first nutrition visit, a significant food allergy was ascertained in one participant; since this represented a protocol violation and safety issue, the participant’s participation was halted, and they were censored from the study, leaving the final study number at 73. Of these, we collected follow-up data at 6 months on 64 (86%) of whom 57 (78%) had complete data on the main outcome (Fig. 1).

Baseline characteristics are shown in Table 1. The mean age of participants was 48 years, 40% were male, 77% Black, 47% had less than a college education, and 49% had household income less than $30,000. Food insecurity was espoused by 51%. The mean A1c was 10.3%, mean BMI was 37.9 kg/m^2^, mean blood pressure was 132.0/78.9 mmHg, and 54% had ≥ 3 medical conditions at baseline.

**Table 1:** Baseline Characteristics of Participants Included in the Analysis (*n* = 57)

Characteristics	Total (*N* = 57)	Intervention (*N* = 30)	Control (*N* = 27)	*p* value^a^
Age in years, mean (SD, range)	47.5 (11.5, 20–66)	44.5 (12.7, 20–62)	50.9 (9.1, 35–66)	0.037
Gender, *n* (%)				0.35
Male	23 (40)	10 (33)	13 (48)	
Female	33 (58)	19 (63)	14 (52)	
Transgender: male to female	1 (2)	1 (3)	0 (0)	
Non-Hispanic ethnicity, *n* (%)	55 (96)	28 (93)	27 (100)	0.49
Race, *n* (%)				0.11
Black/African American	44 (77)	26 (87)	18 (67)	
Others^b^	13 (23)	4 (13)	9 (33)	
Education, *n* (%)				0.21
Less than high school	5 (9)	2 (7)	3 (11)	
High school/GED	22 (38)	9 (30)	13 (48)	
College and above	30 (53)	19 (63)	11 (41)	
Employment, *n* (%)				0.78
Working full-time	17 (30)	9 (30)	8 (30)	
Working part-time	10 (18)	7 (23)	3 (11)	
Unemployed or laid-off	10 (17)	6 (20)	3 (11)	
Not working due to health reasons	13 (23)	5 (17)	8 (30)	
Keeping house/raising children	4 (7)	2 (7)	2 (7)	
Retired	2 (3)	1 (3)	1 (4)	
Other^c^/don’t know	1 (2)	0 (0)	1 (4)	
Income (missing = 14), *n* (%)				0.23
< $30,000 per year	21 (49)	9 (39)	12 (60)	
≥ $30,000 per year	22 (51)	14 (61)	8 (40)	
Low food security, *n* (%)	28 (49)	16 (53)	13 (48%)	0.79
Number of medical conditions, *n* (%)^d^				1.00
< 3 medical conditions	42 (74)	22 (77)	20 (89)	
≥ 3 medical conditions	15 (26)	8 (27)	7 (26)	
Clinical measures				
Mean HbA1c (%) (SD, range)	10.3 (1.9, 8.1–15)	10.2 (2.1, 8.1–15)	10.4 (1.7, 8.1–14.6)	0.73
HbA1c ≥ 9%, *n* (%)	36 (63)	17 (57)	19 (70)	0.41
Mean systolic blood pressure, mm/Hg (SD, range)	132.0 (22.1, 98–227)	129.2 (17.5,106–171)	135.3 (26.4, 98–227)	0.30
Mean diastolic blood pressure, mm/Hg (SD, range)	78.9 (15.6, 52–157)	78.1 (12.2, 57–105)	80.0 (18.9, 52–157)	0.65
Mean BMI, kg/m^2^ (SD, range)	37.9 (10.8, 23.3–66.0)	39.3 (10.8, 23.3–61.0)	36.6 (10.9, 23.8–66.0)	0.36

Data are presented as *N* (%) or mean (SD, range)

^a^Chi^2^ Fisher exact test is used for all characteristics except age and A1c where *t*-test was used

^b^Others include White, Asian, American Indian, mixed

^c^Others included working part-time, unemployed, looking for a job, retired, or not reported

^d^Medical conditions were self-reported and include diabetes, hypertension, high cholesterol, liver or kidney disease, stroke, heart disease, or cancer

### Feasibility and Intervention Delivery

Eighty-six percent of meals were delivered, and the mean number of nutrition visits completed across all participants randomized to the intervention group was 4.8 (of 6 visits), with 41% completing all 6 visits. Food consumption was assessed at three timepoints for each person over the course of the study. At nutrition visits 2 and 3, six of 26 participants who responded (23%) reported eating all or most^9–12^ of the meals provided over the past 7 days, 42% some^5–8^ and 35% few to none (0–4) of the meals. Consumption of food in the produce bag was higher, with 54% reporting use of all or most of the produce in the last week. On average, 72% reported they had never shared food in the last 7 days, and 10% reported sharing it rarely, and 18% shared sometimes or more often. Additionally, 80% reported being satisfied or very satisfied with the meals, and 84% reported being satisfied or very satisfied with the produce bags.

### Outcomes

**Figure 2:**
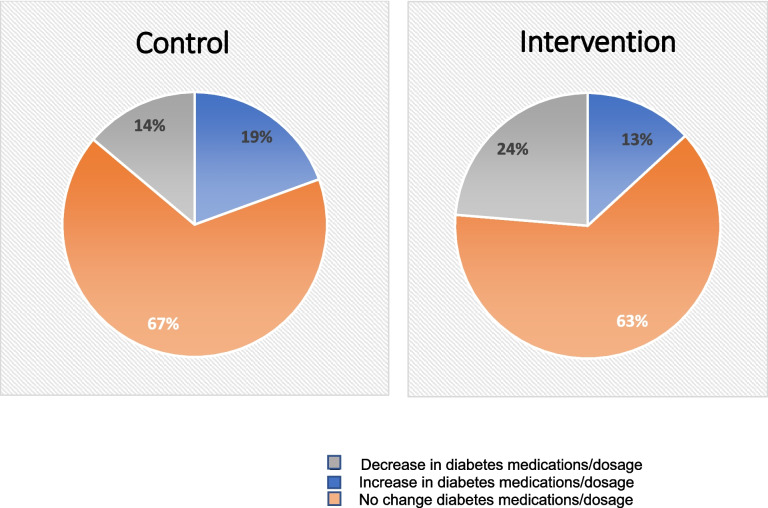
Self-reported change in diabetes medication from baseline to 6 months by study arm

At 6 months, both the intervention and control groups had improvements in the main outcome, A1c (− 0.7 vs. − 0.6%), with no difference between groups (Table 2). In pre-planned exploratory analyses, the control group had more favorable changes in DM medications than the intervention group (Fig. 2). Despite all participants having uncontrolled T2DM at baseline, more than 60% in both groups reported no change in their DM medication over the study period, and a substantial minority (14% and 24%, respectively) reported taking less medication at 6 months compared to baseline. Furthermore, only 9 participants (15.8%) had an A1c checked outside of the study around 12 months after enrollment (± 2 months). There was a trend towards greater improvement in A1c at 6 months among those who reported eating ≥ 9 of the provided meals weekly compared to less (− 1.9% vs. − 0.5%, *p* = 0.14), but no difference in A1c by number of completed nutrition visits (Supplemental Table 2).

**Table 2:** Effect of the FEED Intervention on Primary and Secondary Outcomes

	Intervention				Control				Intervention-control
	*N*^	Mean (SD)	*N*	Difference^+^ (95% CI)	*N*^	Mean (SD)	*N*	Difference^+^ (95% CI)	Difference-in-differences (95% CI)
HbA1c (%)									
Baseline	30	10.2 (2.1)		0.0 (Ref)	27	10.4 (1.7)		0.0 (Ref)	
3-month	24	10.0 (2.1)	24	− 0.3 (− 1.0, 0.4)	23	9.8 (1.7)	23	− **0.6 (**− **1.1,** − **0.13)** *	0.4 (− 0.5, 1.2)
6-month	30	9.5 (1.7)	30	− **0.7 (**− **1.5,** − 0.001) *	27	9.8 (2.0)	27	− 0.6 (− 1.4, 0.1)	− 0.1 (− 1.1, 0.9)
SBP (mmHg)									
Baseline	29	128.2 (17.0)		0.0 (Ref)	25	133.0 (26.1)		0.0 (Ref)	
3-month	23	126.2 (17.1)	23	− 3.3 (− 10.6, 3.9)	21	129.9 (18.6)	21	− 3.9 (− 12.1, 4.1)	0.6 (− 10.0, 11.2)
6-month	28	122.7 (18.1)	28	− **6.3 (**− **12.1,** − 0.6) *	23	129.7 (22.4)	23	− 3.7 (− 10.7, 3.3)	− 2.6 (− 11.4, 6.1)
DBP (mmHg)									
Baseline	29	77.6 (12.2)		0.0 (Ref)	26	79.5 (19.2)		0.0 (Ref)	
3-month	23	76.5 (12.4)	23	− 0.3 (− 5.8, 5.2)	22	80.1 (14.8)	22	0.2 (− 5.2, 5.6)	− 0.5 (− 8.0, 7.0)
6 − month	28	77 (10.2)	28	− 0.8 (− 4.3, 2.6)	24	81.4 (19.5)	24	1.9 (− 2.0, 5.9)	− 2.8 (− 7.9, 2.3)
Weight (lb)									
Baseline	29	234.7 (63.4)		0.0 (Ref)	27	240.4 (74.9)		0.0 (Ref)	
3-month	24	227.3 (65.2)	23	− 1.1 (− 3.7, 1.5)	22	240.2 (82.1)	22	1.2 (− 1.5, 3.9)	− 2.3 (− 5.9, 1.3)
6-month	27	230.2 (62.0)	27	− 0.3 (− 3.1, 2.5)	25	241.1 (80.2)	25	− 1.3 (− 4.9, 2.4)	1.0 (− 3.4, 5.4)
BMI (kg/m^2^)									
Baseline	29	39.3 (10.8)		0.0 (Ref)	27	36.6 (10.9)		0.0 (Ref)	
3-month	23	38.4 (11.6)	23	− 0.2 (− 0.6, 0.2)	22	36.6 (12.0)	22	0.2 (− 0.3, 0.6)	− 0.3 (− 1.0, 0.2)
6-month	27	38.5 (10.6)	27	− 0.02 (− 0.5, 0.5)	25	36.5 (11.7)	25	− 0.2 (− 0.8, 0.3)	0.2 (− 0.5, 0.9)
Quality of life^a^									
Baseline	30	− 4.2 (2.3)		(Ref)	27	− 3.3 (2.8)		(Ref)	
3-month	24	− 4.0 (2.9)	24	− 0.02 (− 0.6, 0.6)	25	− 3.1 (2.2)	25	0.01 (− 0.9, 0.9)	− 0.03 (− 1.1, 1.0)
6-month	29	− 4.0 (2.7)	29	0.1 (− 0.3, 0.5)	27	− 3.4 (2.5)	27	− 0.05 (− 0.7, 0.6)	0.1 (− 0.6, 0.9)
Diabetes self-management^b^									
Baseline	30	20.7 (4.9)		(Ref)	27	20 (6.0)		(Ref)	
3-month	24	23.3 (5.7)	24	3.3 (1.2, 5.3)*	25	20.5 (8.9)	25	0.2 (− 3.2, 3.6)	3.0 (− 0.9, 6.9)
6-month	29	22.5 (7.5)	29	1.8 (− 0.8, 4.3)	27	23.4 (6.6)	27	3.4 (1.2, 5.5)*	− 1.6 (− 4.9, 1.7)
Diet quality^c^									
Baseline	26	44.3 (12.8)		(Ref)	24	50 (10.1)		(Ref)	
3-month	21	47.9 (12)	21	2.6 (− 5.4, 10.5)	21	48.4 (8.8)	21	− 1.5 (− 6.4, 3.4)	4.1 (− 5.0, 13.1)
6-month	22	45.9 (10.9)	22	0.08 (− 4.7, 4.9)	20	48.2 (13.1)	20	− 0.8 (− 7.1, 5.4)	0.9 (− 6.6, 8.5)
Low food security^d^									
Baseline	16	53.3		(Ref)	13	48.1		(Ref)	
3-month	5	16.7	5	− 36.7 (− 36.9, − 36.4)*	12	44.4	12	− 3.7 (− 3.9, − 3.4)*	− 32.9 (− 33.3, − 32.6)*
6-month	9	30.0	9	− 23.3 (− 23.6, − 23.1)*	11	40.7	11	− 7.4 (− 7.7, − 7.1)*	− 15.9 (− 16.3, − 15.6)*

^+^Differences compared by *t*-test. ^Some numbers differ from HbA1c due to missing data or outliers with influence which were removed

^a^Assessed by the Audit of Diabetes-Dependent Quality of Life (ADDQOL); average weighted impact (AWI) scores were used in this comparison

^b^Assessed using the Perceived Diabetes Self-Management Scale (PDSMS)

^c^Assessed using the ASA24 diet recall and calculating Healthy Eating Index Score 2015

^d^Assessed using the USDA’s Household Food Security Questionnaire; differences compared by *t*-test

^*^Significant at *p* < 0.05 level

Similar to A1c, changes in systolic BP (− 6.3 vs. − 3.7 mmHg), diastolic BP (− 0.8 vs. 1.9 mmHg), weight (− 0.3 vs. − 1.3 lbs), and BMI (− 0.02 vs. − 0.20 kg/m^2^) at 6 months did not differ between groups (Table 2, all *p* > 0.05). Food insecurity decreased significantly from baseline to 3 months in the intervention group (53 to 17%) compared to control (48 to 44%; difference-in-differences − 33%, *p* < 0.05), which diminished, but remained statistically significant at 6 months, after meal deliveries had stopped (difference-in-differences − 16%, *p* < 0.05). DM-related quality of life, knowledge, and self-efficacy also improved modestly but not differently across treatment groups (Supplemental Table 2).

Diet quality was low in both groups at baseline (HEI mean score of 44.3 in the intervention group and 50.0 in the control group out of 100; higher scores reflecting better quality)^21^ and not significantly different. Over the first 3 months, the intervention group showed an increase of 2.6 points in HEI, while the control group decreased by 1.5 points, resulting in a positive but not statistically significant difference of 4.1 points between the groups (*p* = 0.37). Notably, there was a significantly different change in wholegrain food intake between the groups (intervention, + 1.7; control, − 1.7; difference, 3.5, *p* = 0.015), but no significant changes were found in other dietary components. There were no significant differences between groups in diet quality at 6 months (Table 2). There was no between-group difference in change in medical costs, either total, or by component, during the 6- or 11-month time windows after start of the intervention, compared to 6 or 11 months prior to enrollment.

## DISCUSSION

In this pilot study of adults participating in Medicaid insurance with T2DM and A1c > 8.0%, we were able to demonstrate that trial enrollment, delivery of MTM for 3 months and up to 6 sessions of MNT over 6 months, and high levels of follow-up were attainable. However, the intervention did not improve A1c levels, blood pressure, weight, or BMI compared to usual care. We did show improvement in food insecurity levels among those receiving the MTM at 3 months, which was diminished but sustained after meal delivery ceased. Analyses of dietary intake also suggested some positive changes overall, with significant increases in whole grain intake at 3 months, which was not sustained. Review of reported medication data suggests that relative increases in medications for diabetes in the control group compared to the intervention group may have accounted for the modest A1c improvements in that arm, perhaps resulting in the lack of difference between study arms. Additional exploration suggested that those who ate more of the delivered foods had greater improvements in A1c levels.

Overall, two-thirds of patients whose A1c was over 8% at baseline did not report any increases in their medication over 6 months, and about 20% reported taking less medication. Furthermore, at 12 months after enrollment, A1c was checked by participants’ regular primary care clinicians in only 16% of people (9 of 57). These findings suggest that the overall quality of diabetes care these participants received during the study period was low. While there are likely many factors for this, which were beyond the scope of this study, it seems clear in retrospect that delivery of MTM for 3 months and MNT for 6 sessions over 6 months in an accessible fashion was likely helpful, but not sufficient to improve the outcomes we had specified.

To date, data on the clinical effects of provision of food, including MTM, on outcomes in patients with T2DM is limited, and study quality has been low.^22^ Prior studies of MTM in patients with uncontrolled T2DM have shown improvement in dietary quality,^14,16^ food security, and diabetes self-management, but typically not clinical outcomes. Berkowitz et al. conducted a randomized cross-over trial of 12 weeks of home-delivered MTM compared to usual care with a Choose MyPlate brochure in adults with T2DM.^14^ They found a statistically significant 31-point higher HEI score when people were getting the meals, as well as less hypoglycemia and lower food insecurity. They did not show any difference in the A1c, lipids, blood pressure, or BMI outcomes. Palar et al. conducted a pre-post analysis of an existing 6-month MTM food and grocery pick-up program for patients with low income and HIV, which expanded to serve patients with DM.^16^ Data collected on 72% of those getting the intervention showed significant improvements in food security, as well as reduced diabetes distress, increased perceived diabetes self-management but no significant changes in A1c levels or A1c < 7%, though the latter showed a trend (10.3 to 19.2%, *p* = 0.08). Notably, they saw a marked decrease in hospitalizations and ED visits over a 3-month period post-intervention compared to before the intervention in both the intervention and control groups (15.7 to 5.8% and 25.0 to 6.9%, respectively) which did not reach statistical significance (both *p* = 0.09). Finally, while food pick-up was high (93%), over 80% of individuals reported throwing away food or sharing food regularly, and 58% reported doing this at least once a week.

There are a number of plausible explanations for the lack of effect of MTM and MNT on clinical outcomes in our study. First, while delivery of meals was high, the limited data we collected on consumption of meals suggests that only about a quarter of participants (8 of 30 reporting) ate all the meals provided. Even if fully consumed, the delivered meals and produce provided an estimated 50–60% of the total food intake each week; it is possible the rest of the food consumed included unhealthy foods. This study took place during the beginning and height of the COVID-19 pandemic which impacted both food variety in the meal intervention and access to food more broadly in populations from which study participants were drawn. Moveable Feast had to reduce menu variety due to staffing and supply chain constraints for a significant portion of the intervention period. This may have led to reduced intake related to fatigue of receiving the same menu items. At the same time, individual level access to food was at a crisis point rarely seen at this scale in society. This may have impacted the types of food consumed in addition to MTM or led to the sharing of food within a household.

The study has several limitations. The meals were tailored to be compliant with American Heart Association recommendations for people with T2DM, and were carbohydrate controlled at a static level for all participants (< 60 g of carbohydrate per meal), and not tailored to each individual. This may have represented a higher carbohydrate intake for some participants since the carbohydrate level was not able to be tailored based on personal characteristics such as height, weight, and sex. Still, this likely represented an improvement in diet quality overall, which should have helped improve diabetes and hypertension control. The MNT was self-directed and did not encompass a specific T2DM self-management curriculum; consistent with the Moveable Feast approach, it was focused on individual goal-setting and general education about nutrition, carbohydrates, physical activity, and diabetes-friendly approaches to diet. It is possible that a more structured, diabetes-focused curriculum and approach would have resulted in improved diabetes knowledge and self-efficacy, and improved A1c levels. As noted above, most patients did not have their diabetes medications adjusted despite having high A1c levels, and many were not getting recommended A1c monitoring. Due to COVID-19, we did not reach our enrollment goal, and thus there is less precision to our effect size estimate. Furthermore, the pandemic may have affected access to usual care, including follow-up visits for medication adjustment and A1c testing. Finally, while the population enrolled generally reflects those enrolled with this Medicaid provider, we did not compare directly and there are likely differences between those who enrolled and those who did not.

The study has several notable strengths. Our strong partnerships with a community-based organization (Moveable Feast) and Medicaid provider (Priority Partners MCO) resulted in reasonable recruitment and good retention of our target population and high delivery of both the MTM and MNT intervention components. Rates of data collection, especially for our primary outcome, were high. Outcome assessment at 6 and 12 months is sufficiently long to be clinically meaningful for both T2DM and hypertension, as well as costs and utilization measures.

In conclusion, provision of MTM and MNT to Medicaid-insured adults with uncontrolled T2DM improved food security but did not result in meaningful improvements in A1c levels, blood pressure, or patient-reported outcomes. Our study confirms that the quality of care such patients are receiving is low, and supports the need for a more comprehensive, multi-component and multi-level approach to care that encompasses multiple socioecological domains (e.g., behavioral, physical environment, sociocultural environment, and health care system) and multiple levels of influence (from individual to societal).^23^ More comprehensive and clinically integrated interventions are likely needed to achieve significant clinical benefits.

## Supplementary Information

Below is the link to the electronic supplementary material.

Supplementary file1 (DOCX 28 KB)

## Data Availability

Data are available by request to the corresponding author.
