# Supplementary material for: Impact of Medically Tailored Meals on Clinical Outcomes Among Low-Income Adults with Type 2 Diabetes: A Pilot Randomized Trial
Source: J Gen Intern Med. 2024 Dec 13;40(8):1711–9. doi: 10.1007/s11606-024-09248-x (PMC12120087; doi:10.1007/s11606-024-09248-x)
Supplement: Supplementary file 1 — Supplementary file1 (DOCX 28 KB) [file 11606_2024_9248_MOESM1_ESM.docx]

# Supplemental Tables

## Supplemental Table 1. Baseline Characteristics of All Randomized Participants (n=74)

| **Characteristics** | **Total**  **(N=74)** | **Intervention**  **(N=38)** | **Control**  **(N=36)** | **P value^a^** |
| --- | --- | --- | --- | --- |
| **Age in years, mean (SD, Range)** | 47.7 (11, 20-66) | 45.6 (12.3, 20-63) | 49.9 (9.1, 35-66) | 0.09 |
| **Gender, n (%)** |  |  |  | 1.00 |
| Male | 30 (41) | 15 (39) | 15 (42) |  |
| Female | 43 (58) | 22 (58) | 21 (58) |  |
| Transgender: Male to Female) | 1 (1) | 1 (3) | 0 (0) |  |
| **Non-Hispanic Ethnicity, n (%)** | 72 (97) | 36 (95) | 36 (100) | 0.49 |
| **Race, n (%)** |  |  |  | 0.39 |
| Black/African American | 59 (80) | 32 (84) | 27 (75) |  |
| Others^b^ | 15 (20) | 6 (15) | 9 (25) |  |
| **Education, n (%)** |  |  |  | 0.53 |
| Less than high school | 6 (8) | 3 (8) | 3 (8) |  |
| High school/GED | 30 (41) | 13 (34) | 17 (47) |  |
| College and above | 38 (51) | 22 (58) | 16 (44) |  |
| **Employment, n (%)** |  |  |  | 0.89 |
| Working full-time | 22 (30) | 11 (29) | 11 (31) |  |
| Working part-time | 10 (14) | 7 (18) | 3 (8) |  |
| Unemployed or laid-off | 15 (20) | 8 (21) | 7 (19) |  |
| Not working due to health reasons | 20 (27) | 9 (24) | 11 (31) |  |
| Keeping house /raising children | 4 (5) | 2 (5) | 2 (6) |  |
| Retired | 2 (3) | 1 (3) | 1 (3) |  |
| Other^c^ /Don’t know | 1 (1) | 0 (0) | 1 (3) |  |
| **Income (missing=16), n (%)** |  |  |  | 0.60 |
| < $30,000 per year | 34 (59) | 17 (55) | 17 (63) |  |
| ≥ $30,000 per year | 24 (41) | 14 (42) | 10 (37) |  |
| **Number of medical conditions, n (%)^d^** |  |  |  | 0.80 |
| < 3 medical conditions | 52 (70) | 26 (68) | 26 (72) |  |
| ≥ 3 medical conditions | 22 (30) | 12 (32) | 10 (28) |  |
| **Clinical Measures** |  |  |  |  |
| Mean HbA1c (%) (SD, Range) | 10.4  (1.9, 8.1-15) | 10.2  (1.9, 8.1-15) | 10.5  (1.8, 8.1-14.6) | 0.49 |
| HbA1c ≥ 9%, n (%) | 49 (66) | 24 (63) | 25 (69) | 0.63 |
| Mean Systolic Blood Pressure, mm/Hg (SD, Range) | 131.5  (21.6, 98-227) | 130.3  (20.0,103-198) | 132.8  (23.4, 98-227) | 0.62 |
| Mean Diastolic Blood Pressure, mm/Hg (SD, Range) | 79.3  (14.2, 52-157) | 78.5  (11.8, 57-105) | 80.2  (16.5, 52-157) | 0.61 |
| Mean BMI, kg/m^2^ (SD, Range) | 36.8  (10.1, 23.3-66.0) | 37.4  (10.5, 23.3-61.0) | 36.3  (9.7, 23.8-66.0) | 0.66 |
| Data are presented as N (%) or mean (SD, range)  ^a^ Chi2 Fisher exact test is used for all characteristics except Age and A1c where t-test was used.  ^b^ Others include White, Asian, American Indian, mixed.  ^c^ Others included working part-time, unemployed, looking for a job, retired, or not reported.  ^d^ Medical conditions were self-reported and include: diabetes, hypertension, high cholesterol, liver or kidney disease, stroke, heart disease, or cancer. | | | | |

## Supplemental Table 2: Mean Change in A1C from Baseline to 3 and 6 Months in the Intervention and Control Groups Stratified by Key Characteristics and Intervention Adherence Measures

|  |  | **3 months** | |  |  | **6 months** | |  |
| --- | --- | --- | --- | --- | --- | --- | --- | --- |
| **Baseline Characteristic** | **N^a^** | **Intervention** | **Control** | **P value**† | **N** | **Intervention** | **Control** | **P value^b^** |
| **Gender** |  |  |  | 0.05 |  |  |  | 0.35 |
| **Female** | 27 | 0.4 (1.1) | -0.6 (1.0) |  | 33 | -0.4 (1.4) | -0.7 (1.5) |  |
| **Male** | 20 | -1.3 (1.8) | -0.7 (1.3) |  | 24 | -1.3 (2.6) | -0.6 (2.1) |  |
| **Race** |  |  |  | 0.47 |  |  |  | 0.15 |
| **Black** | 37 | -0.4 (1.6) | -0.7 (1.2) |  | 44 | -0.9 (2.0) | -0.4 (1.9) |  |
| **Non-Black** | 10 | 0.6 (1.6) | -0.4 (1.1) |  | 13 | 0.3 (1.2) | -1.1 (1.4) |  |
| **Employment** |  |  |  | 0.71 |  |  |  | 0.21 |
| **Full-time** | 14 | -0.8 (1.9) | -0.9 (1.3) |  | 17 | -1.8 (2.1) | -0.7 (1.7) |  |
| **Other than full-time** | 33 | 1.1 (1.4) | -0.5 (1.1) |  | 40 | -0.3 (1.8) | -0.62 (1.9) |  |
| **Household Income^c^** |  |  |  | 0.49 |  |  |  | 0.13 |
| **> $30,000/year** | 16 | -0.7 (2.1) | -1.3 (0.5) |  | 22 | -0.9 (1.5) | -1.3 (1.2) |  |
| **≤ $30,000/year** | 17 | -0.2 (1.6) | -0.1 (1.2) |  | 21 | -1.2 (2.8) | 0.2 (1.6) |  |
| **Missing** | 14 | 0.4 (0.6) | -0.8 (1.1) |  | 14 | 0.2 (1.1) | -1.3 (2.1) |  |
| **Food Insecurity** |  |  |  | 0.87 |  |  |  | 0.80 |
| **Insecure** | 23 | -0.5 (1.7) | -0.8 (1.2) |  | 29 | -0.8 (2.1) | -0.6 (1.9) |  |
| **Secure** | 24 | -0.1 (1.6) | -0.4 (1.0) |  | 28 | -0.7 (1.8) | -0.7 (1.7) |  |
| **Baseline A1C** |  |  |  | 0.07 |  |  |  | 0.03 |
| **<9%** | 18 | 0.8 (0.8) | -0.2 (0.9) |  | 21 | 0.8 (1.2) | -0.1 (1.2) |  |
| **≥9 %** | 29 | -0.2 (1.6) | -0.8 (01.2) |  | 36 | -2.0 (1.5) | -0.9 (1.7) |  |
| **Intervention Group Only** |  |  |  |  |  |  |  |  |
| **Number of meals eaten per week** |  |  |  | 0.14 |  |  |  | 0.14 |
| **< 9** | 18 | -0.1 ±0.3 | - |  | 21 | -0.5 ±0.4 |  |  |
| **≥9** | 5 | -1.3 ±0.9 | - |  | 5 | -1.9 ±0.9 |  |  |
| **Number of produce bag used per week** |  |  |  | 0.92 |  |  |  | 0.24 |
| **All/most/Some** | 15 | -0.4±0.5 | - |  | 19 | -0.5 ±0.4 |  |  |
| **Very little/none** | 8 | -0.3±0.4 | - |  | 7 | -1.6±0.8 |  |  |
| **Number of nutrition visits completed (out of 6)** |  |  |  |  |  |  |  | 0.96 |
| **<5** |  | - | - |  | 9 | -0.7 ±0.9 |  |  |
| **≥ 5** |  | - | - |  | 21 | -0.8 ±0.3 |  |  |
| Values shown are mean ±SEs.  ^a^ At 3 months only 47 participants had an A1c measured; ^b^ P-values calculated using an interaction analysis for each characteristic separately using linear regression; ^c^ 14 participants did not report their income | | | | | | | | |

**Supplemental Table 3: Study Outcomes and Covariates Assessed at 0, 3, 6, and 12 month follow-up**

| **Measure** | **Assessment Type** | **Assessment Month** | | | |
| --- | --- | --- | --- | --- | --- |
|  |  | 0 | 3 | 6 | 12 |
| Sociodemographics | Questionnaire | X |  |  |  |
| Comorbidities | Questionnaire/Claims | X | X | X |  |
| Dietary intake | ASA24 diet recall Questionnaire | X | X | X |  |
| Food insecurity | USDA’s Household Food Security Questionnaire | X | X | X |  |
| Medications | Questionnaire | X | X | X |  |
| Quality of Life | Audit of Diabetes-Dependent Quality of Life (ADDQOL) | X | X | X |  |
| Self-efficacy | Perceived Diabetes Self-Management Scale (PDSMS) | X | X | X |  |
| Blood Pressure | Physical exam | X | X | X |  |
| Weight | Physical exam | X | X | X |  |
| Hemoglobin A1c | Abbott Afinion AS100 Analyzer machine | X | X | X | X |
| Health care utilization | Claims data | X | X | X | X |
| Health care costs | Claims data | X | X | X | X |
